# Arthropathie destructrice des épaules au cours d'une acromégalie

**DOI:** 10.4314/pamj.v10i0.72220

**Published:** 2011-10-01

**Authors:** Nessrine Akasbi, Latifa Tahiri, Ouafae Lyhyaoui, Mohammed Elidrissi, Ghita Sqalli Houssaini, Abdelmajid Elmrini, Farida Ajdi, Harzy Taoufik

**Affiliations:** 1 Service de Rhumatologie, CHU Hassan II, Fès, Maroc; 2 Service d'Endocrinologie, CHU Hassan II, Fès, Maroc; 3 Service de Chirurgie ostéo-articulaire B4, CHU Hassan II, Fès, Maroc

**Keywords:** Arthropathie, acromégalie, épaules, Maroc

## Abstract

L'acromégalie est une maladie endocrinienne rare, en rapport avec une hypersécrétion d'hormone de croissance. Elle a des conséquences rhumatologiques: l'arthropathie périphérique, l'atteinte rachidienne et les syndromes canalaires. L'atteinte articulaire accompagne une acromégalie active, sa survenue après un traitement radical et une rémission complète est rare. Nous présentons le cas d'une patiente de 70 ans ayant un antécédent d'acromégalie sur adénome hypophysaire il y a 25 ans, traitée chirurgicalement et déclarée en rémission complète, a développé une arthropathie destructrice des deux épaules. Le but de notre observation est de mettre le point sur la possibilité d'une atteinte articulaire au cours de l'acromégalie et de son retentissement fonctionnelle.

## Introduction

L'acromégalie est maladie endocrinienne rare, qui peut affecter le squelette osseux sous l'effet de l'hormone de croissance. Nous rapportons l'observation d'une patiente de 70 ans ayant un antécédent d'acromégalie il y a 25 ans, traitée chirurgicalement et déclarée en rémission complète, qui a développé une arthropathie destructrice des deux épaules.

## Patient et observation

**Figure 1:**
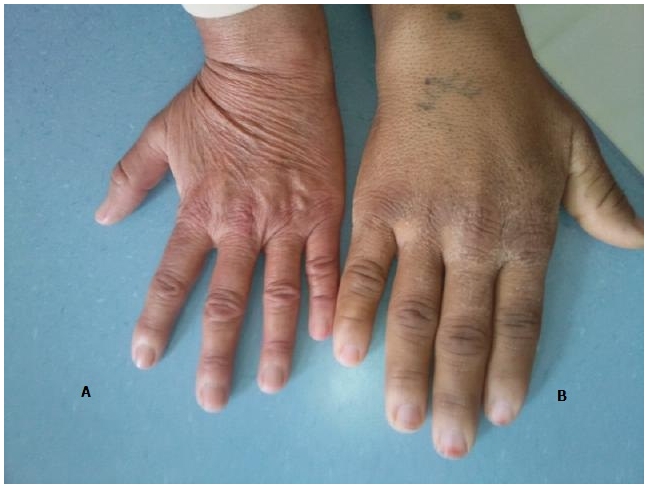
La main de la patiente atteinte d'acromégalie (B) comparée à la main d'une femme normale (A)

**Figure 2:**
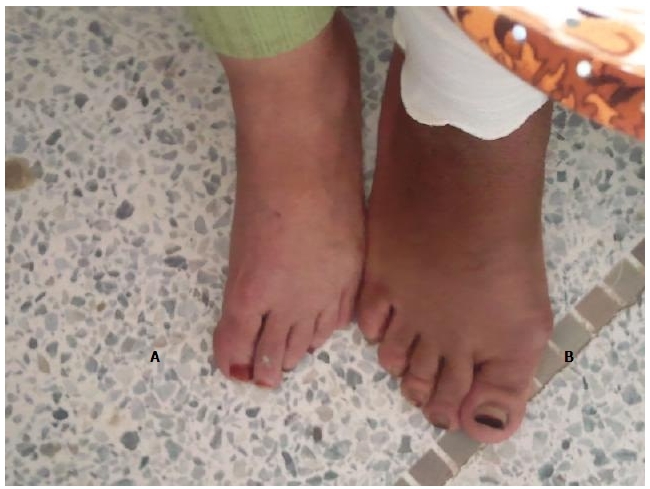
Le pied de la patiente atteinte d'acromégalie (B) comparé à un pied d'une femme saine (A)

Patiente âgée de 70 ans, ayant un antécédent d'acromégalie (Figure 1 et Figure 2) sur adénome hypophysaire il y a 25 ans, confirmée sur les données cliniques, biologiques et radiographiques, traitée chirurgicalement et déclarée en rémission complète. Elle a présenté depuis 5 ans des scapulalgies mécaniques bilatérales d'aggravation progressive avec une limitation fonctionnelle majeure. On a noté chez la patiente un nez épaté, des pommettes saillantes, un front bombé, des lèvres épaisses, des rides marquées ainsi que des doigts et des orteils boudinés, séquelles de son acromégalie.

**Figure 3:**
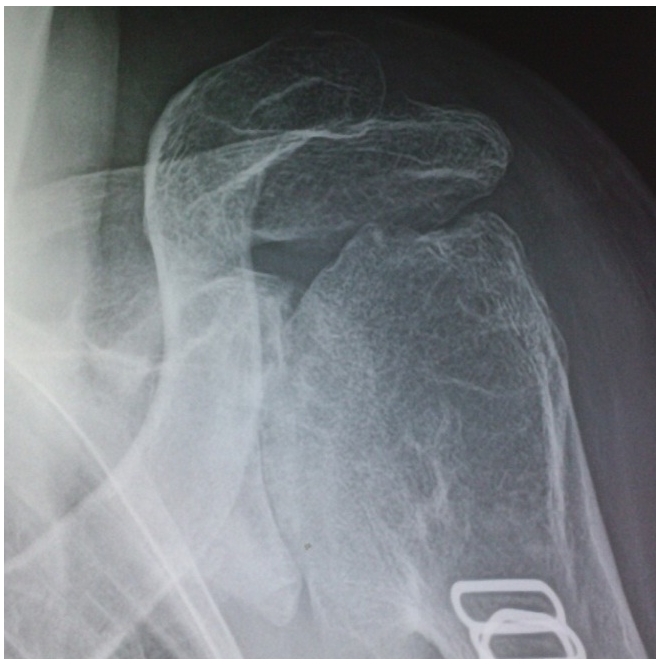
Radiographie de l’épaule de la patiente atteinte d'acromégalie montrant une arthropathie destructrice

L'examen ostéo-articulaire a trouvé des épaules douloureuses et limitées en rotation interne (20°), rotation externe (20°), antépulsion (90°) et abduction (80°) en bilatérale, sans signe inflammatoire en regard. La radiographie des deux épaules a montré une arthropathie destructrice bilatérale des épaules avec un pincement total de l'interligne articulaire, la présence de géodes, d'ostéophytes exubérants, et des exostoses avec des hypertrophies osseuses diffuses (Figure 3). Les radiographies des autres sites articulaires et du rachis étaient sans particularités. Le bilan biologique n'a pas objectivé de syndrome inflammatoire (VS: 12 mm, CRP: 4 mg/l), ou de perturbation du bilan phosphocalcique (Ca: 90 mg/l, Ph: 25 mg/l, PAL: 85 UI/l). Le bilan immunologique (Facteur rhumatoïde, anticorps anti CCP, anticorps anti nucléaires et anti ECT) était négatif. L’échographie des deux épaules n'a pas objectivé de bursite inflammatoire ou dégénérative ou de tendinopathie des coiffes des rotateurs.

Le diagnostic d'une arthropathie acromégalique destructrice des deux épaules apparu après un traitement radical, a été retenu. La patiente a refusé le remplacement prothétique des épaules, un traitement palliatif a été proposé à base d'antalgique, de rééducation et de physiothérapie.

## Discussion

L'acromégalie est une maladie rare, habituellement en rapport avec une hypersécrétion d'hormone de croissance (GH) par un adénome hypophysaire. Les manifestations ostéo-articulaires liées à l'acromégalie sont rares. Elles regroupent l'atteinte articulaire périphérique, axiale et canalaire [1]. Ce sont la principale cause de morbidité et de limitation fonctionnelle retentissant sur la qualité de vie des patients.

L'atteinte articulaire au cours de l'acromégalie touche préférentiellement les hanches, les épaules, les genoux et les coudes parfois les mains et les pieds.

Les mécanismes physiopathologiques incriminés sont le retentissement de l'hypersécrétion de GH sur l'os et ses conséquences mécaniques, ainsi que l'hypersécrétion locale en intra-articulaire d'insuline growth factor I (IGF-I) ce qui entraîne une hypertrophie et une hyperplasie ostéo-cartilagineuse et ligamentaire irréversible, à l'origine de la limitation articulaire [2].

L'arthropathie acromégalique peut être parmi les premiers symptômes de la maladie, et apparait au cours d'une acromégalie active sous l'effet de l'hormone de croissance. La prévalence et la sévérité de l'atteinte articulaire surtout destructrice sont associées à la durée d'exposition aux sécrétions de GH incontrôlées et réfractaires au traitement. L'atteinte est double, articulaire et axiale. Les arthralgies sont généralement de rythme mécanique. À un stade évolué, la mobilité articulaire peut être limitée due à l'arthropathie destructrice qui est une forme rare comme c'est le cas chez notre patiente [3].

Sur le plan radiologique, on note un pincement des interlignes articulaires, des ostéophytes exubérants, des ossifications des insertions tendineuses et des exostoses [4].

Bluestone et al. [5] a montré dans une étude portée sur 42 patients ayant une acromégalie que dans certains cas l'atteinte articulaire apparait même après un traitement radical et que le rôle et l'efficacité du traitement de l'hypersécrétion de GH dans le contrôle des symptômes rhumatologiques sont parfois aléatoires et variables d'un patient à un autre.

Chez notre patiente, l'apparition de l'atteinte articulaire destructrice était après un traitement radical et une rémission complète de 25 ans, témoignait que le retentissement des secrétions d'hormone de croissance sur les segments ostéo-articulaires est irréversible et que le traitement chirurgical n’était pas efficace sur la prévention de l'atteinte rhumatologique.

Le traitement en phase de début de l'hypersécrétion hormonale permet une récupération de l'atteinte articulaire. Il fait appel, en générale, à plusieurs thérapeutiques sur l'adénome: une résection ou même une radiothérapie, ainsi à des thérapeutiques sur l'atteinte articulaire périphérique, notamment des antalgiques, des anti-inflammatoires et des infiltrations locales de corticothérapie ou parfois un remplacement prothétique avec une rééducation pré et post-opératoire [5].

## Conclusion

L'arthropathie acromégalique est rare. Le but de notre observation est de mettre le point sur la possibilité d'une arthropathie destructrice au cours de l'acromégalie qui reste exceptionnelle. C'est la principale cause de morbidité et de réduction de la qualité de vie. Un traitement radical sur l'hypersécrétion de GH peut ne pas prévenir l'apparition d'une atteinte ostéo-articulaire.
